# Mechanisms Underlying Negative p53 Immunocytochemistry in Oral Cytology: *TP53* mRNA Expression and Sampling Limitations

**DOI:** 10.3390/diagnostics16172811

**Published:** 2026-09-01

**Authors:** Takayuki Miyakawa, Satoshi Maruyama, Manabu Yamazaki, Tatsuya Abé, Shigehiro Ono, Kei Tomihara, Jun-ichi Tanuma

**Affiliations:** 1 Division of Oral Pathology, Faculty of Dentistry & Graduate School of Medical and Dental Sciences, Niigata University, 2-5274 Gakkocho-dori, Chuo-ku, Niigata 951-8514, Japan; president@ltanhouse.com (T.M.); manyamaz@dent.niigata-u.ac.jp (M.Y.); abet@dent.niigata-u.ac.jp (T.A.); 2 Oral Pathology Section, Department of Surgical Pathology, Niigata University Hospital, 1-754 Asahimachi-dori, Chuo-ku, Niigata 951-8520, Japan; maru@dent.niigata-u.ac.jp; 3 Division of Reconstructive Surgery for Oral and Maxillofacial Region, Faculty of Dentistry & Graduate School of Medical and Dental Sciences, Niigata University, 2-5274 Gakkocho-dori, Chuo-ku, Niigata 951-8514, Japan; shigehiro.ono.8020@niigata-u.ac.jp; 4 Division of Oral and Maxillofacial Surgery, Faculty of Dentistry & Graduate School of Medical and Dental Sciences, Niigata University, 2-5274 Gakkocho-dori, Chuo-ku, Niigata 951-8514, Japan; tomihara@dent.niigata-u.ac.jp

**Keywords:** oral liquid-based cytology, *TP53* mRNA, p53 immunocytochemistry, oral high-grade squamous intraepithelial lesion, oral squamous cell carcinoma

## Abstract

**Background:** Reliable molecular biomarkers that complement cytomorphological assessment for early identification of high-risk oral epithelial lesions remain limited. Although p53 immunocytochemistry (ICC) is used as a surrogate marker of *TP53* abnormalities, p53 protein expression does not always reflect *TP53* transcriptional activity. This study investigated the relationships between p53 ICC, *TP53* mRNA expression, and p53 immunohistochemical (IHC) staining patterns in the categories of oral high-grade squamous intraepithelial lesions (OHSILs) and oral squamous cell carcinoma (SCC). **Methods:** We classified 504 liquid-based cytology (LBC) specimens according to the Bethesda System for Reporting Oral Cytology: negative for an intraepithelial lesion or malignancy (NILM) (*n* = 394), oral low-grade squamous intraepithelial lesion (OLSIL) (*n* = 72), OHSIL (*n* = 18), and SCC (*n* = 20). p53 ICC was performed on cytological specimens, *TP53* mRNA expression was quantified by quantitative real-time PCR using residual LBC samples, and corresponding biopsy specimens were evaluated for p53 IHC staining patterns. Associations between p53 ICC and clinicopathological variables were also analyzed. **Results:** The p53 labeling index significantly increased with cytological severity, and *TP53* mRNA expression was significantly higher in OHSIL and SCC than in NILM and OLSIL. In OHSIL and SCC, lesions showing a null-type p53 IHC staining pattern showed significantly lower *TP53* mRNA expression than lesions with non-null staining patterns. No significant associations between p53 ICC and clinicopathological variables were observed in OHSIL. In contrast, p53 ICC positivity in SCC was significantly associated with tumor size, depth of invasion, and p53 IHC staining patterns. **Conclusions:** Negative p53 ICC may result from sampling limitations or null-type *TP53* expression. *TP53* mRNA analysis provides complementary molecular information that distinguishes false-negative p53 ICC caused by sampling limitations from biologically reduced *TP53* expression. Integrating cytomorphology, p53 ICC, histopathology, and *TP53* mRNA analysis may improve diagnostic accuracy and risk stratification of high-risk oral epithelial lesions.

## 1. Introduction

Oral squamous cell carcinoma (SCC) is one of the most common malignancies of the head and neck region and is frequently preceded by oral potentially malignant disorders (OPMDs), including oral epithelial dysplasia (OED). The patient’s prognosis is strongly associated with the stage at diagnosis. Therefore, early detection of high-risk lesions remains a major clinical challenge.

Historically, to improve the standardization and diagnostic accuracy of oral cytology in Japan, the Japanese Society of Clinical Cytology published domestic diagnostic guidelines in 2015 [1]. Building upon these efforts, the diagnostic criteria for oral cytology were subsequently revised to promote more standardized reporting and risk stratification, leading to the introduction of a Bethesda System-based reporting framework in Japan in 2022 [2,3]. This framework established standardized diagnostic categories, including oral low-grade squamous intraepithelial lesion (OLSIL) and oral high-grade squamous intraepithelial lesion (OHSIL), and recommended liquid-based cytology (LBC) for specimen preparation.

Despite these advances, reliable molecular biomarkers that complement cytomorphological assessment and accurately identify high-risk oral epithelial lesions remain limited. In daily practice, distinguishing reactive epithelial atypia from an OHSIL or early SCC based solely on cytomorphological features may be difficult. Consequently, cytopathologists and oral pathologists often encounter challenges in assessing the biological significance and malignant potential of atypical lesions.

Among the molecular biomarkers investigated to date, p53 is one of the most widely used and clinically accepted markers for evaluating oral epithelial lesions. *TP53* is the most frequently altered tumor suppressor gene in head and neck squamous cell carcinoma, with genomic studies demonstrating *TP53* alterations in approximately 72% of HNSCCs and in up to 84% of HPV-negative tumors [4]. *TP53* alterations represent an early event in oral carcinogenesis and are frequently detected in OPMDs and OSCC [5,6,7,8]. Therefore, p53 has been extensively investigated as a biomarker for the early detection and risk assessment of oral epithelial lesions [5,6].

Immunohistochemistry (IHC) and immunocytochemistry (ICC) using anti-p53 antibodies are routinely performed in pathology laboratories and are widely used as surrogate methods for evaluating *TP53* abnormalities [7]. However, p53 protein expression does not always reflect *TP53* transcriptional activity or the mutation-associated biological status. In particular, lesions showing a null immunostaining pattern may retain *TP53* mRNA expression despite the absence of detectable p53 protein, whereas other lesions completely lack *TP53* mRNA and p53 protein expression. Furthermore, exfoliative oral cytology is inherently subject to sampling limitations because genetically altered cells may remain confined to the basal and parabasal epithelial layers. Consequently, p53 ICC alone may underestimate the molecular abnormalities associated with high-risk oral epithelial lesions.

Recent advances in quantitative real-time PCR (qRT-PCR) have enabled highly sensitive and quantitative assessment of *TP53* mRNA expression in clinical specimens [7]. Unlike ICC, which detects protein accumulation, qRT-PCR directly measures *TP53* transcriptional activity and may thus provide additional molecular information regarding p53-null lesions. Evaluation of *TP53* mRNA expression may help clarify the biological heterogeneity of p53-null lesions and improve the molecular characterization of oral epithelial lesions.

This study aimed to investigate the relationships between p53 ICC expression, *TP53* mRNA expression, and p53 IHC staining patterns in the categories of OHSIL and SCC diagnosed according to the Bethesda System for Reporting Oral Cytology. By integrating p53 ICC, quantitative *TP53* mRNA analysis using qRT-PCR, and histopathological findings, we aimed to clarify the biological characteristics of p53-null lesions and to identify the two principal mechanisms underlying negative p53 ICC results in oral cytology, with particular emphasis on *TP53* mRNA expression and sampling limitations.

## 2. Materials and Methods

### 2.1. Patients and Study Design

This retrospective study included patients who underwent oral LBC at Niigata University Medical and Dental Hospital between January 2023 and April 2026. Cytological diagnoses were classified according to the Bethesda System for Reporting Oral Cytology as negative for an intraepithelial lesion or malignancy (NILM), OLSIL, OHSIL, and SCC. In cases for which biopsy specimens were available, histopathological diagnoses were used as the reference standard. *TP53* mRNA expression analysis was performed using residual LBC specimens from NILM, OLSIL, OHSIL, and SCC cases.

This study was approved by the Ethics Committee of Niigata University (Approval No. G2022-0027) and was conducted in accordance with the Declaration of Helsinki.

### 2.2. Histopathological Examination

Biopsy specimens were fixed in 10% neutral-buffered formalin, embedded in paraffin, sectioned at 4 μm, and stained with hematoxylin and eosin. Histological diagnoses were established independently by two experienced oral pathologists according to the WHO Classification of Head and Neck Tumours [8].

### 2.3. p53 ICC

Oral LBC specimens were prepared using the SurePath^®^ liquid-based cytology system (BD Diagnostics, Franklin Lakes, NJ, USA). To perform p53 immunocytochemistry, separate slides were prepared from residual SurePath LBC specimens rather than using destained Papanicolaou-stained slides or cell-block preparations. ICC was performed using a mouse monoclonal anti-p53 antibody (clone DO-7; Dako/Agilent Technologies, Glostrup, Denmark). Antigen retrieval was carried out in EDTA buffer (pH 8.0) using microwave heating, followed by incubation with the primary antibody for 2 h at room temperature. Immunoreactivity was visualized using the EnVision™ detection system (Dako, Glostrup, Denmark) with 3,3′-diaminobenzidine as the chromogen.

The p53 labeling index (LI) was calculated as the percentage of p53-positive atypical epithelial cells among at least 500 evaluated epithelial cells. Cytological specimens containing distinct nuclear staining in atypical epithelial cells were regarded as p53-positive.

### 2.4. p53 IHC

IHC was performed on formalin-fixed paraffin-embedded biopsy specimens using the same antibody (clone DO-7) and staining protocol as that used for ICC. According to the distribution of p53-positive cells, staining patterns were classified into four categories using the following criteria proposed by Sawada et al. [5]: normal (NM), basal/parabasal (BP), high-expression (HI), and loss (LS). All slides were independently evaluated by two oral pathologists.

### 2.5. RNA Isolation and qRT-PCR

Total RNA was extracted from the residual cell suspension remaining in the BD SurePath™ LBC collection vial (Becton, Dickinson and Company, Franklin Lakes, NJ, USA) after preparation of cytological slides using the RNAqueous^®^ Kit (Ambion, Grand Island, NY, USA) according to the manufacturer’s instructions. The RNA concentration and purity were assessed using a NanoDrop Lite UV–Vis spectrophotometer (Thermo Fisher Scientific, Wilmington, DE, USA). RNA integrity was not directly assessed using the RNA integrity number or fragment analysis before cDNA synthesis. After reverse transcription, the concentration of the synthesized cDNA was also determined using the same instrument [9,10,11].

The cDNA was synthesized from total RNA using the High-Capacity cDNA Reverse Transcription Kit (Applied Biosystems, Foster City, CA, USA). We performed qRT-PCR using TaqMan™ Universal PCR Master Mix (Applied Biosystems) and TaqMan Gene Expression Assays for *TP53* (Assay ID: Hs01034249_m1). We used 18S rRNA (Hs99999901_s1) as the endogenous control for normalization.

Relative *TP53* mRNA expression levels were calculated using the 2^−ΔΔCt^ method. *TP53* mRNA expression in OLSIL, OHSIL, and SCC samples was normalized to that in NILM specimens, which served as the calibrator. All reactions were performed in triplicate, and the mean values were used for statistical analysis. Relative *TP53* mRNA expression was further compared between cases with a null-type p53 IHC staining pattern and those with non-null p53 IHC staining patterns in the OHSIL and SCC categories.

### 2.6. Statistical Analysis

Continuous variables are presented as the mean ± standard deviation. Comparisons of the p53 LI and *TP53* mRNA expression between cytological diagnostic categories were performed using one-way analysis of variance followed by Tukey’s multiple-comparison test. Associations between p53 ICC findings and clinicopathological variables were analyzed separately in the OHSIL and SCC categories using Fisher’s exact test. The Fisher–Freeman–Halton exact test was applied for variables containing more than two categories.

All statistical analyses were performed using EZR software (Jichi Medical University Saitama Medical Center, version 1.33). All tests were two-sided, and *p*-values < 0.05 were considered statistically significant [12].

## 3. Results

### 3.1. Clinicopathological Characteristics

A total of 510 oral LBC specimens were collected during the study period. Six specimens classified as inadequate were excluded, with 504 satisfactory specimens remaining for analysis. Of these, 394 were classified as NILM, 72 as OLSIL, 18 as OHSIL, and 20 as SCC (Table 1).

A histopathological examination was available in 154 cases, comprising 44 NILM, 72 OLSIL, 18 OHSIL, and 20 SCC cases. Among the 18 OHSIL cases, histological diagnoses included SCC in 11 (61.1%) cases, severe OED or carcinoma in situ (CIS) in 6 (33.3%) cases, and moderate OED in 1 (5.6%) case. Among the 20 SCC cases, 16 (80.0%) were histologically confirmed as SCC and 4 (20.0%) as severe OED/CIS.

No p53-positive cases were identified in NILM or OLSIL. In contrast, p53 ICC positivity was detected in 2 of 18 (11.1%) OHSIL cases and in 13 of 20 (65.0%) SCC cases (Table 2).

### 3.2. p53 ICC Expression in Oral Epithelial Lesions

**Figure 1:**
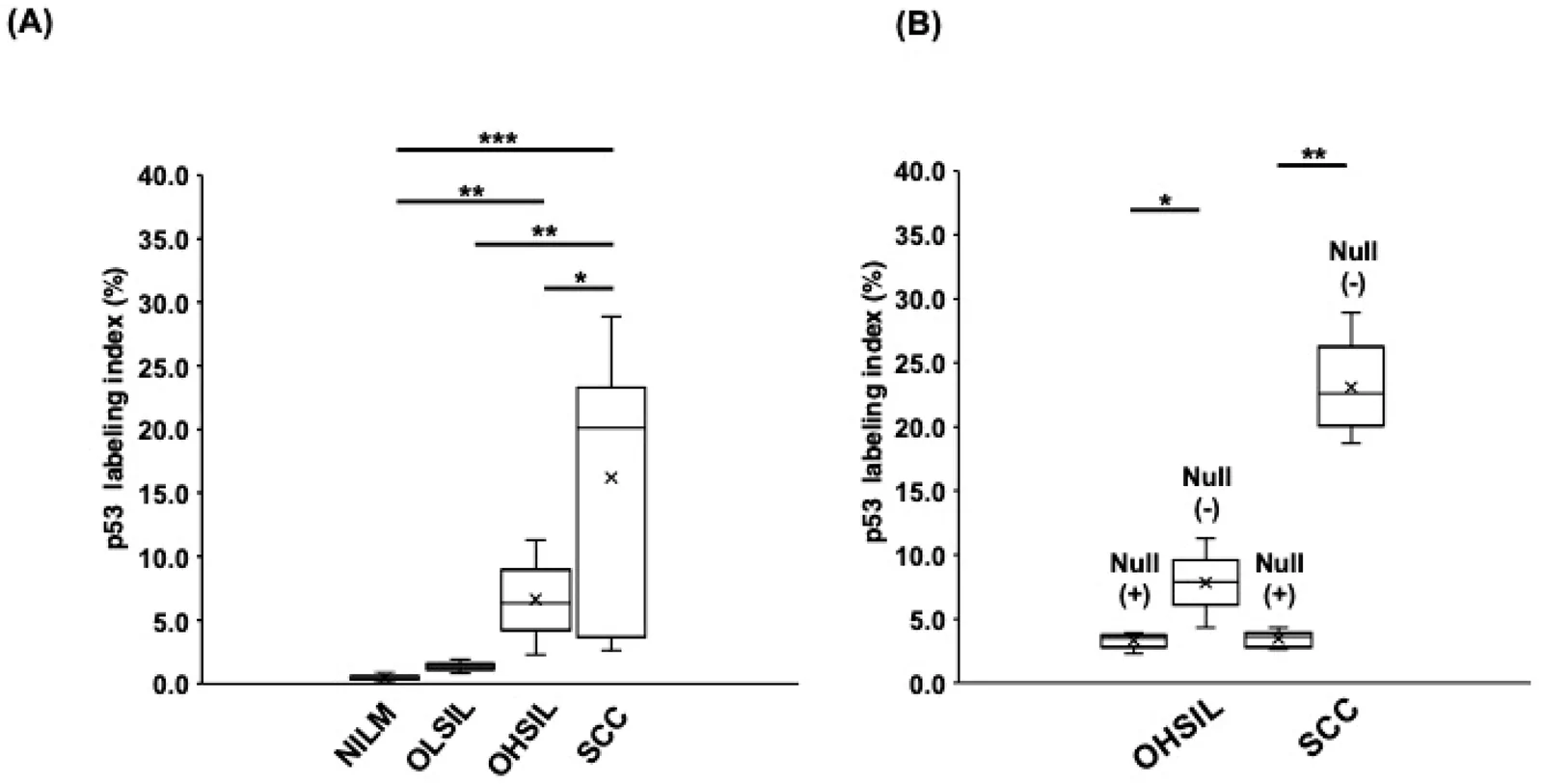
(**A**) Relationship between cytological diagnosis and the p53 labeling index. The p53 labeling index (LI) progressively increased with cytological severity. The proportion of p53-positive cases and the mean p53 LI were significantly higher in the categories of oral high-grade squamous intraepithelial lesion (OHSIL) and squamous cell carcinoma (SCC) than in negative for an intraepithelial lesion or malignancy (NILM) and oral low-grade squamous intraepithelial lesion (OLSIL). These findings indicate an association between p53 accumulation and increasing cytological severity. (**B**) p53 LI according to the presence or absence of a null-type p53 IHC staining pattern in OHSIL and SCC. Cases showing a null-type p53 IHC staining pattern [Null (+)] showed significantly lower p53 labeling indices than cases with non-null p53 IHC staining patterns [Null (−)] in OHSIL and SCC. These findings indicate that lesions with a null-type p53 IHC staining pattern are characterized by markedly reduced p53 protein expression in cytological specimens. The boxes indicate the IQR, the horizontal line represents the median, whiskers indicate the minimum and maximum values, and × denotes the mean. *p*-values were calculated using one-way analysis of variance followed by Tukey’s multiple-comparison test. * *p* < 0.05, ** *p* < 0.01, *** *p* < 0.001.

The p53 LI progressively increased according to the cytological severity (Figure 1A). The proportion of p53-positive cases and the mean p53 LI were significantly higher in OHSIL and SCC than in NILM and OLSIL, indicating an association between p53 accumulation and increasing cytological severity. The p53 labeling index was further compared according to the presence or absence of a null-type p53 IHC staining pattern (Figure 1B). Cases showing a null-type p53 IHC staining pattern showed significantly lower p53 labeling indices than cases with a non-null p53 IHC staining pattern in OHSIL and SCC. These findings indicate that lesions with a null-type p53 IHC pattern generally show markedly reduced or absent p53 protein expression in cytological specimens.

### 3.3. TP53 mRNA Expression According to the Presence or Absence of a Null-Type p53 IHC Staining Pattern

**Figure 2:**
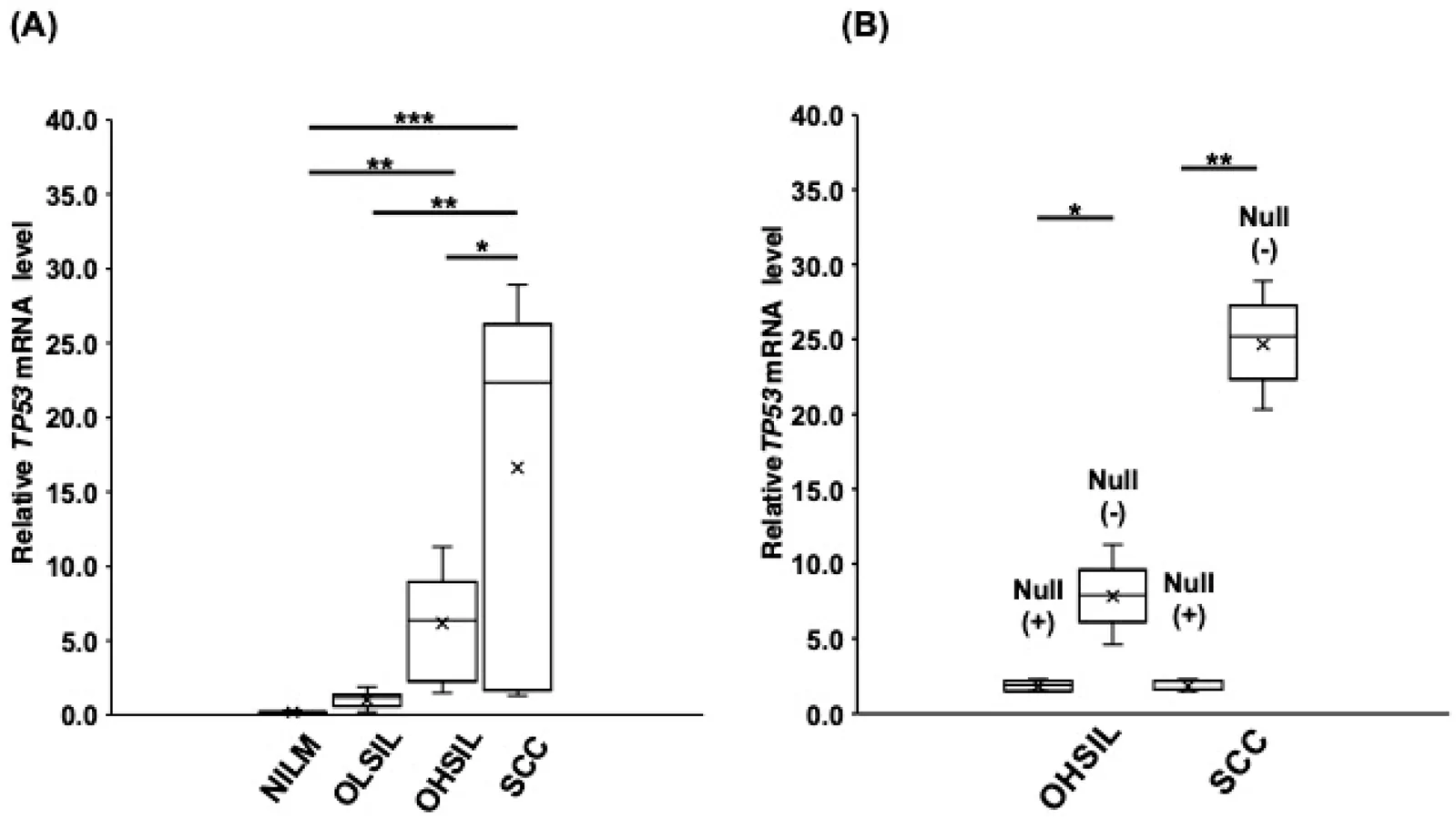
(**A**) Relative *TP53* mRNA expression according to cytological diagnosis. Relative *TP53* mRNA expression was quantified by qRT-PCR in oral LBC specimens classified as negative for NILM, OLSIL, OHSIL, or SCC. *TP53* mRNA expression progressively increased with the cytological severity and was significantly higher in OHSIL and SCC than in NILM and OLSIL. The boxes indicate the IQR, the horizontal line represents the median, whiskers indicate the minimum and maximum values, and × denotes the mean. Statistical significance was determined using one-way analysis of variance followed by Tukey’s multiple-comparison test. * *p* < 0.05, ** *p* < 0.01, *** *p* < 0.001. (**B**) Relative *TP53* mRNA expression according to the presence or absence of a null-type p53 IHC staining pattern in OHSIL and SCC. Relative *TP53* mRNA expression was compared between lesions with a null-type p53 IHC staining pattern [Null (+)] and those with non-null p53 IHC staining patterns [Null (−)] separately in OHSIL and SCC cases. In OHSIL and SCC, lesions with a null-type p53 IHC staining pattern showed significantly lower *TP53* mRNA expression than lesions with a non-null p53 IHC staining pattern. These findings suggest that the null-type p53 IHC staining pattern is associated with reduced TP53 transcription. The boxes indicate the IQR, the horizontal line represents the median, whiskers indicate the minimum and maximum values, and × denotes the mean. * *p* < 0.05, ** *p* < 0.01.

Relative *TP53* mRNA expression progressively increased with cytological severity and was significantly higher in OHSIL and SCC than in NILM and OLSIL (Figure 2A). *TP53* mRNA expression was further compared between lesions with a null-type p53 IHC staining pattern and those with non-null p53 IHC staining patterns separately in the OHSIL and SCC categories (Figure 2B).

In OHSIL and SCC, lesions with a null-type p53 IHC staining pattern showed significantly lower *TP53* mRNA expression than lesions with a non-null p53 IHC staining pattern. These findings suggest that the null-type p53 IHC staining pattern is associated with reduced *TP53* transcription.

### 3.4. Representative p53 IHC Staining Patterns

**Figure 3:**
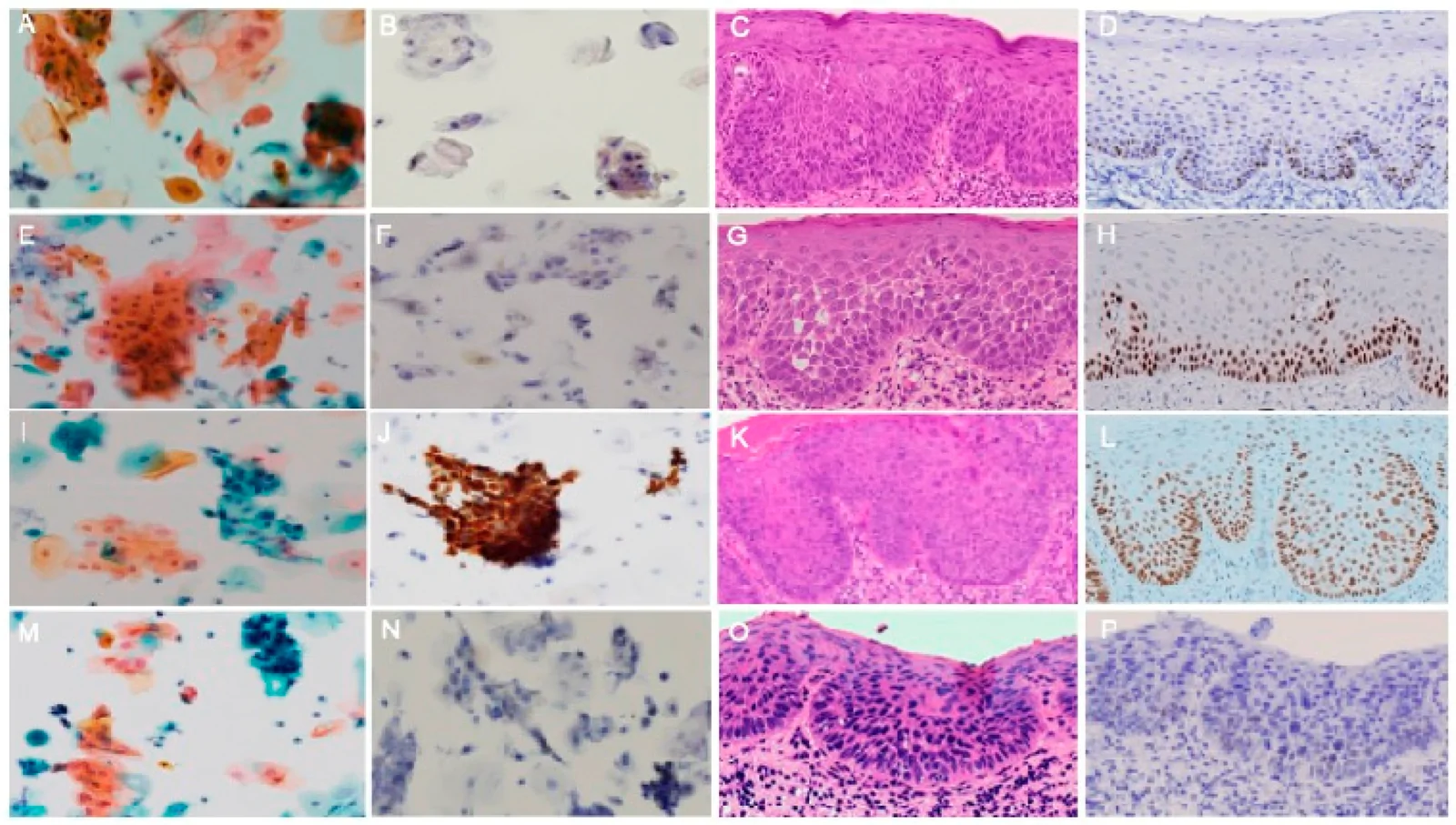
Representative cytological, histological, and IHC findings of the four p53 staining patterns in OHSIL and SCC. Representative Papanicolaou-stained LBC, p53 ICC, hematoxylin and eosin, and p53 IHC images illustrating the four p53 staining patterns. (**A**–**D**) Normal pattern: scattered p53-positive cells restricted to the basal or parabasal layers. (**E**–**H**) Basal/parabasal pattern: diffuse p53-positive cells involving the basal and parabasal layers. (**I**–**L**) High-expression pattern: diffuse p53-positive cells extending into the suprabasal layers. (**M**–**P**) Loss pattern (also referred to as null-type): complete absence of p53 immunoreactivity despite preserved epithelial morphology.

**Figure 4:**
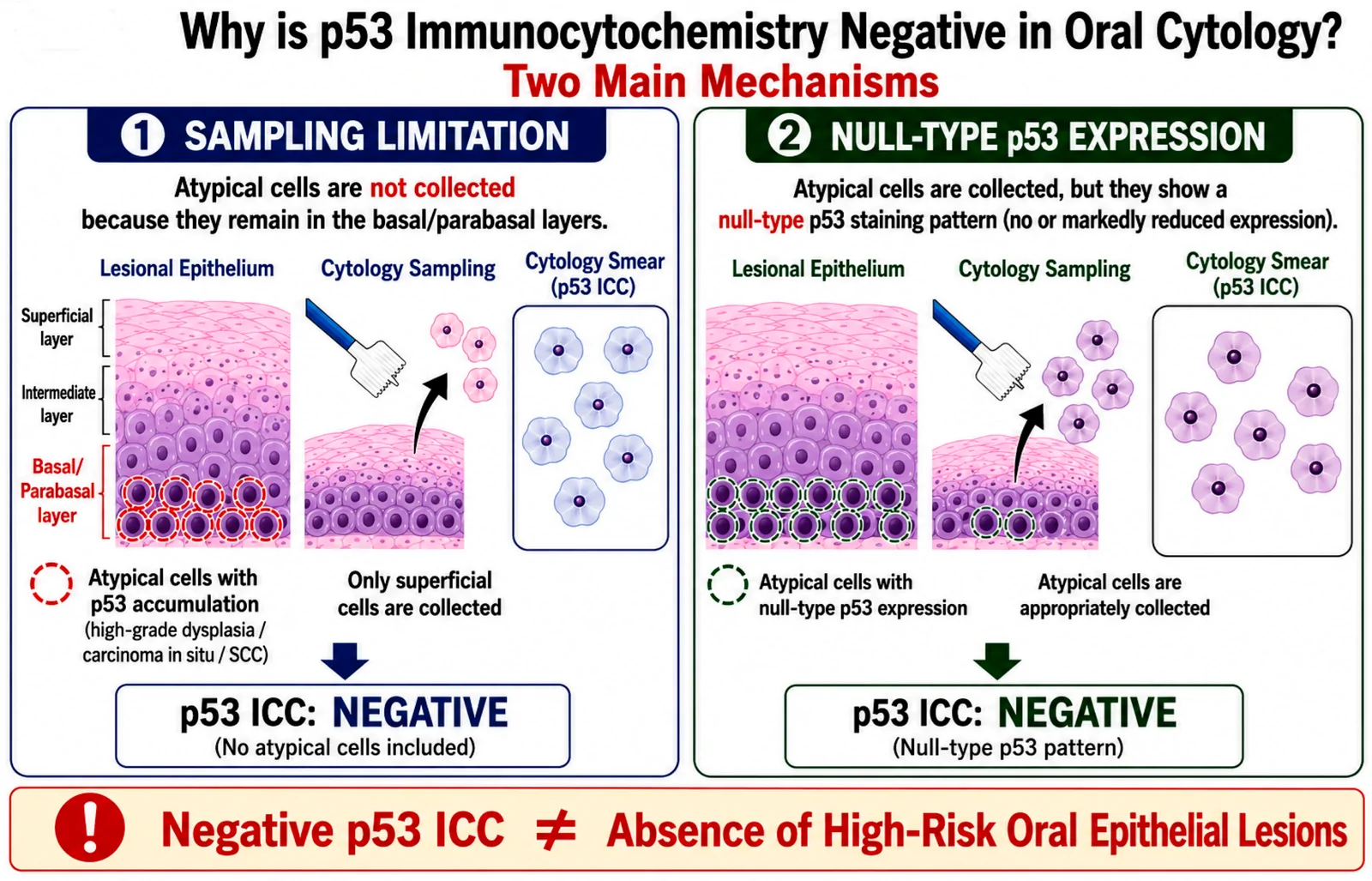
Two hypotheses to explain the mechanisms underlying negative p53 ICC in oral cytology. Negative p53 ICC in oral cytology may result from two biologically distinct mechanisms. (**Left panel**) Sampling limitations: atypical epithelial cells remain confined to the basal/parabasal layers and are not recovered by exfoliative cytology. Consequently, only superficial epithelial cells are collected, resulting in false-negative p53 ICC despite the presence of high-risk epithelial lesions. (**Right panel**) Null-type p53 expression: atypical epithelial cells are successfully collected, but they show a null-type p53 staining pattern because of a complete absence or marked reduction in p53 protein expression, resulting in negative p53 ICC despite adequate cellular sampling. Taken together, these mechanisms provide a conceptual framework for interpreting negative p53 immunocytochemistry in oral cytology.

Representative cytological, histological, ICC, and IHC findings of the four p53 staining patterns are shown in Figure 3. The biological interpretation of these findings is shown in Figure 4, which illustrates the two principal mechanisms responsible for negative p53 ICC in oral cytology. Negative p53 ICC may result from sampling limitations, in which atypical cells are not recovered, or from biologically reduced p53 protein expression associated with the null-type staining pattern. The NM and BP patterns showed p53-positive cells confined to the basal or parabasal layers, whereas the HI pattern showed diffuse staining extending into the suprabasal layers. In contrast, the LS (also referred to as null-type) pattern demonstrated complete absence of p53 immunoreactivity despite preservation of epithelial architecture.

### 3.5. Associations Between p53 ICC Expression and Clinicopathological Factors

The associations between p53 ICC positivity and clinicopathological variables are shown in Table 2. In patients with an OHSIL (*n* = 18), no clinicopathological variables showed significant associations with p53 ICC positivity. Specifically, there was no significant association between tumor size and p53 ICC positivity (*p* = 0.497), and similar results were observed for the depth of invasion (*p* = 0.497).

In contrast, in patients with SCC (*n* = 20), p53 ICC positivity was significantly associated with the tumor size (*p* = 0.004), greater depth of invasion (DOI) (*p* = 0.031), and p53 IHC staining pattern (*p* < 0.001). Specifically, the p53 ICC positivity rate was significantly higher in tumors measuring ≥20 mm (91.7%, 11/12 cases) than in those measuring <20 mm (25.0%, 2/8 cases). Similarly, SCCs with a DOI ≥ 5 mm showed a significantly higher p53 ICC positivity rate (80.0%, 12/15 cases) than those with a DOI < 5 mm (20.0%, 1/5 cases). Regarding the p53 IHC staining patterns, all SCCs showing the HI pattern (10/10) were positive for p53 ICC, whereas all SCCs showing the LS pattern (4/4) were negative for p53 ICC. In contrast, p53 ICC-positive and p53 ICC-negative cases were observed in SCC with the NM and BP staining patterns.

## 4. Discussion

The principal finding of this study was that negative p53 ICC resulted from two biologically distinct mechanisms: sampling limitations and null-type p53 expression. By integrating p53 ICC, *TP53* mRNA expression, and p53 IHC staining patterns, we found that lesions with a null-type p53 IHC staining pattern showed significantly lower *TP53* mRNA expression than lesions with a non-null p53 IHC staining pattern. These findings indicate that the null-type p53 IHC pattern is associated with reduced *TP53* transcription, supporting the concept that the LS-type p53 IHC staining pattern represents a distinct molecular subgroup characterized by reduced *TP53* expression. Furthermore, these findings support the complementary use of *TP53* mRNA analysis for interpreting negative p53 ICC results in high-risk oral epithelial lesions.

The early detection of malignant transformation in OPMDs remains one of the greatest challenges in oral pathology. Although the introduction of the Japanese Society of Clinical Cytology guidelines and the Bethesda System for Reporting Oral Cytology has markedly improved the standardization and diagnostic accuracy of oral cytology [1,2,3], cytomorphological assessment alone remains insufficient for accurately identifying lesions with malignant potential. Previous studies have shown that the incorporation of molecular biomarkers into LBC improves the diagnostic performance and facilitates the detection of early oral SCC [2,3,7]. Our findings further emphasize that molecular assessment is particularly useful when p53 ICC shows negative results despite morphologically suspicious lesions.

*TP53* is the most frequently altered tumor suppressor gene in head and neck squamous cell carcinoma and represents one of the earliest genetic events during oral carcinogenesis [4,6]. Consequently, p53 IHC and ICC have been widely used as surrogate markers of *TP53* abnormalities [5,13,14,15,16]. However, these techniques evaluate only protein accumulation and do not necessarily reflect *TP53* transcriptional activity.

In this study, the p53 LI progressively increased from NILM and OLSIL to OHSIL and SCC, consistent with previous reports [2,5,7,14,15]. This progressive increase supports the concept that *TP53*-abnormal epithelial cells gradually expand from the basal and parabasal layers toward the epithelial surface during oral carcinogenesis [4,6]. As the proportion of abnormal cells increases, exfoliative cytology is more likely to capture these cells, explaining the substantially higher p53 positivity observed in SCC than in OHSIL.

Our findings indicate that negative p53 ICC should not be interpreted as representing a single mechanism but rather two biologically distinct mechanisms (Figure 4). The first mechanism is sampling limitation, in which atypical epithelial cells remain confined to the basal or parabasal layers and are thus not recovered by exfoliative cytology. This phenomenon is particularly relevant in OHSIL, where genetically altered cells frequently occupy only the lower epithelial layers. The second mechanism is null-type p53 expression, in which atypical cells are successfully collected but show complete absence or marked reduction in p53 protein expression, resulting in negative p53 immunostaining despite adequate sampling. Distinguishing these two mechanisms is clinically important because both of them show negative p53 ICC findings but reflect fundamentally different biological processes. Accordingly, negative p53 ICC should always be interpreted in conjunction with cytomorphological findings, histopathological evaluation, and *TP53* mRNA analysis whenever available.

Another important finding of this study was the concordant reduction in *TP53* mRNA expression and p53 labeling indices in lesions showing a null-type p53 IHC staining pattern. Nevertheless, p53 ICC should be interpreted cautiously because the sensitivity of exfoliative cytology depends on adequate recovery of *TP53*-abnormal cells. In OHSIL, in particular, *TP53*-abnormal cells are frequently confined to the basal and parabasal epithelial layers, limiting their exfoliation into cytological specimens and increasing the likelihood of false-negative p53 ICC. Therefore, negative p53 ICC should not be interpreted as evidence that *TP53*-abnormal cells are absent.

Sawada et al. [5] demonstrated that p53 IHC staining patterns accurately predict the *TP53* mutation status in OED and proposed the NM, BP, HI, and LS patterns as useful pathological indicators. Our findings further extend these observations by showing that the LS pattern was accompanied by reduced *TP53* mRNA expression [15,16]. In this study, p53 ICC positivity was significantly associated with the p53 IHC staining pattern. While all HI pattern SCCs were p53 ICC-positive and all LS pattern SCCs were p53 ICC-negative, positive and negative ICC findings occurred in the NM and BP patterns. These findings suggest that p53 ICC generally reflects the biological characteristics of p53 IHC staining patterns but may still be affected by sampling variability, particularly in lesions with limited numbers of *TP53*-abnormal cells [17]. Therefore, integrating p53 IHC with *TP53* mRNA analysis provides complementary molecular information for the evaluation of high-risk oral epithelial lesions.

The contrasting findings between OHSIL and SCC are consistent with the biological progression of oral carcinogenesis. In OHSIL, *TP53*-abnormal cells are frequently confined to the basal and parabasal epithelial layers, limiting their recovery by exfoliative cytology and reducing the sensitivity of p53 ICC. Consequently, p53 ICC findings should be interpreted with caution because the diagnostic performance of exfoliative cytology depends on adequate recovery of *TP53*-abnormal cells [18]. As lesions progress to SCC, expansion of *TP53*-abnormal cell populations increases the likelihood of exfoliation into cytological specimens, thereby improving the sensitivity of p53 ICC. This biological progression is consistent with the significant associations observed between p53 ICC positivity and a larger tumor size, greater DOI, and p53 IHC staining patterns in SCC. These findings suggest that false-negative p53 ICC results are more likely to occur in early lesions, particularly OHSIL, than in advanced invasive SCC.

Our findings have direct implications for cytopathological practice. A negative p53 ICC result should not preclude a biopsy when cytological atypia is present but should be interpreted together with cytomorphological and, where available, histopathological findings. In selected high-risk cases, *TP53* mRNA analysis provides complementary molecular information that distinguishes true biological loss of *TP53* expression from false-negative p53 ICC caused by sampling limitations. The presence of p53 ICC-positive and p53 ICC-negative cases in the NM and BP p53 IHC staining patterns further indicates that p53 ICC results should always be interpreted together with histopathological findings rather than in isolation. Such an integrated diagnostic approach may reduce false-negative interpretation and improve risk stratification of OHSIL and early SCC [14]. Accordingly, integrating cytomorphology, p53 ICC, histopathology, and *TP53* mRNA analysis may improve the evaluation of high-risk oral epithelial lesions beyond p53 ICC alone. Notably, 11 of the 18 (61.1%) OHSIL cases were subsequently diagnosed as invasive SCC in a histopathological examination. This relatively high proportion may reflect the inherent limitation of exfoliative cytology in assessing stromal invasion because cytology primarily evaluates cells collected from the epithelial surface. Therefore, high-grade intraepithelial lesions and superficially invasive SCC may show overlapping cytomorphological features. In addition, targeted biopsies from clinically suspicious areas may detect focal invasion not represented in cytological specimens. These findings highlight the importance of a histopathological examination for the definitive assessment of stromal invasion.

This study had several limitations. First, the relatively small number of OHSIL cases (*n* = 18) may have reduced the statistical power to detect associations between p53 immunocytochemical findings and clinicopathological parameters. Therefore, the findings in the OHSIL subgroup should be interpreted with caution. A post hoc power analysis was not performed because of the retrospective and exploratory nature of the study. Validation in larger, preferably multicenter cohorts is necessary to confirm the reproducibility and generalizability of these findings. Second, only *TP53* mRNA expression was investigated, whereas other molecular alterations associated with oral carcinogenesis were not evaluated. Third, although the RNA concentration and purity were assessed spectrophotometrically, RNA integrity was not directly evaluated using the RNA integrity number or fragment analysis. Therefore, partial RNA degradation associated with fixation and storage of residual SurePath LBC specimens cannot be completely excluded as a technical variable. Previous studies have shown that fixation and storage conditions of LBC specimens can affect RNA integrity and downstream molecular analyses [19,20,21]. The inherent limitations of RNA preservation in LBC fixatives may affect the amplifiability of long transcripts or lower-abundance mRNAs in some samples. To improve the technical reliability of the qRT-PCR analysis, relative *TP53* mRNA expression was normalized using 18S rRNA as an endogenous control, and all qRT-PCR reactions were performed in triplicate.

## 5. Conclusions

Negative p53 immunocytochemistry is the result of two distinct mechanisms: sampling limitations and null-type p53 expression. Therefore, negative p53 ICC should not be interpreted as evidence of the absence of a high-risk oral epithelial lesion. Integrating cytomorphology, p53 ICC, histopathology, and *TP53* mRNA analysis may improve diagnostic accuracy and reduce false-negative interpretation. These findings provide a practical framework for interpreting negative p53 ICC in the cytological evaluation of high-risk oral epithelial lesions.

## Figures and Tables

**Table 1:** Clinicopathological characteristics and histopathological findings according to oral cytological classification. Biopsy-confirmed cases with p53-positive cytology rate (%). 0 (0/44) *; p53-positive rate among biopsy-confirmed cases.

Cytological Category (The Bethesda System)	NILM	OLSIL	OHSIL	SCC
Subclass (Japanese class classification)	I–II	IIb–III	IIIb–IV	V
Total Cases	394	72	18	20
Histological diagnosis for biopsy (Total)	44	72	18	20
SCC	1	5	11	16
Severe OED/CIS	1	15	6	4
Moderate OED	2	20	1	0
Mild OED	4	20	0	0
Epithelial hyperplasia	3	5	0	0
Lichen planus	1	1	0	0
Papilloma	1	2	0	0
Verruciform xanthoma	1	2	0	0
No malignancy	30	2	0	0
Cytology p53-positive rate (%)	0	0	11.1	65.0
	(0/44) *	(0/72)	(2/18)	(13/20)

**Table 2:** Association between p53 ICC expression and clinicopathological characteristics in OHSIL and SCC.

	OHSIL (*n* = 18)			SCC (*n* = 20)		
	p53 ICC			p53 ICC		
	(+)	(−)	*p*-Value	(+)	(−)	*p*-Value
**Age**						
≤60	2	6	0.680	4	2	0.963
60>	0	10		9	5	
**Gender**						
Male	2	11	1.000	7	3	1.000
Female	0	5		6	4	
**Locations**						
Tongue	2	8	0.615	6	1	0.407
Gingiva	0	4		4	2	
Buccal mucosa	0	2		2	3	
Others	0	2		1	1	
**Tumor size** (mm)						
<20	0	7	0.497	2	6	0.004
≥20	2	9		11	1	
**Depth of Invasion** (DOI: mm)						
<5	0	7	0.497	2	5	0.031
≥5	2	9		11	2	
**Histological diagnosis**						
Moderate OED	0	1	0.111	0	0	0.584
Severe OED/CIS	0	6		4	0	
SCC	2	9		9	7	
**p53-IHC patterns**						
NM	0	5	0.209	2	2	<0.001
BP	0	6		1	1	
HI	2	0		10	0	
LS	0	5		0	4	

**Note:** No p53-positive cases were identified among NILM or OLSIL cases; p53 positivity was observed only in OHSIL and SCC cases. **Abbreviations:** NM, normal pattern; BP, basal/parabasal pattern; HI, high-expression pattern; LS, loss pattern (also referred to as null-type).

## Data Availability

The datasets used and/or analyzed during the current study are available upon reasonable request from the corresponding author.
